# Supplementary material for: Infection Dynamics of Swine Influenza Virus in a Danish Pig Herd Reveals Recurrent Infections with Different Variants of the H1N2 Swine Influenza A Virus Subtype
Source: Viruses. 2020 Sep 10;12(9):1013. doi: 10.3390/v12091013 (PMC7551734; doi:10.3390/v12091013)
Supplement: Supplementary file 1 [file viruses-12-01013-s001.pdf]

viruses

Infection dynamics of swine influenza virus in a Danish pig herd reveals recurrent infections with different variants of the H1N2 swine influenza A virus subtype

Tarka Raj Bhatta [1,2,3,*], Pia Ryt-Hansen [3], Jens Peter Nielsen [3], Lars Erik Larsen [3], Inge Larsen [3], Anthony Chamings [1,2], Nicole B. Goecke [3,4] and Soren Alexandersen [1,2,5,*]

[1] Geelong Centre for Emerging Infectious Diseases, Geelong, VIC 3220, Australia
[2] Deakin University, Geelong, VIC 3220, Australia
[3] University of Copenhagen, Department of Veterinary and Animal Sciences, Frederiksberg C, Denmark
[4] Division for Diagnostics & Scientific Advice, National Veterinary Institute, Technical University of Denmark, Lyngby, Denmark
[5] Barwon Health, University Hospital Geelong, Geelong, VIC 3220 Australia.
* Correspondence: trbhatta@deakin.edu.au (T.R.B.); soren.alexandersen@deakin.edu.au (S.A.); Tel.: +61-0-342159635 (S.A.)

Table 1. Occurrence and recurrence of IAV in pigs from week 2 to week 22 in the pig herd using high-throughput rtPCR. '+' indicates the positive case of IAV whereas '-' indicates the negative case of IAV. Green boxes indicate the non-consecutive detection of IAV, yellow boxes indicate the consecutive detection of IAV whereas blue boxes indicate the single detection of IAV. 'NA' indicate the unavailability of the sample for IAV detection test.

Pig sample ID	Week 2	Week 3	Week 4	Week 5	Week 6	Week 8	Week 12	Week 22
10	-	-	+	-	-	+	-	+
20	-	NA	NA	-	-	-	-	NA
30	-	NA	-	-	-	-	-	NA
40	-	NA	-	-	-	-	NA	NA
50	-	NA	NA	-	-	NA	NA	NA
60	-	-	-	+	+	-	-	NA
70	-	+	-	+	+	+	-	NA
80	-	-	+	-	+	-	-	NA
90	-	-	+	-	-	-	-	-
100	-	-	+	+	+	NA	NA	NA
110	-	-	-	-	+	-	-	-
120	NA	-	-	-	+	-	-	-
130	+	-	-	-	-	-	-	NA
140	-	-	-	-	-	-	+	NA
150	NA	NA	NA	NA	NA	NA	NA	NA
160	-	-	-	-	-	-	-	NA
170	-	+	-	-	-	+	-	NA
180	-	-	-	+	-	-	-	NA
190	-	NA	NA	NA	NA	NA	NA	NA
200	-	NA	NA	-	-	-	-	NA
210	-	-	-	-	NA	+	-	-
220	NA	NA	NA	NA	NA	NA	NA	NA
230	-	-	-	-	+	-	-	NA
240	-	-	-	-	-	-	-	NA
250	-	-	+	-	-	+	-	-
260	-	-	-	-	-	-	-	NA
270	-	-	+	-	-	-	-	NA
280	-	-	+	+	-	-	-	NA
290	-	-	-	+	+	-	-	NA
300	-	+	-	-	-	-	-	NA
310	-	-	+	NA	NA	NA	NA	NA
320	NA	NA	NA	NA	NA	NA	NA	NA
330	-	-	NA	-	+	-	-	NA
340	-	-	-	+	+	+	-	NA

350	-	-	-	-	-	-	-	-
360	-	-	-	+	-	-	-	NA
370	-	-	-	+	-	-	-	-
380	-	-	+	+	-	-	-	+
390	-	-	-	+	+	-	-	+
400	-	-	-	+	-	-	-	NA
410	-	NA	NA	+	-	-	-	-
420	-	-	-	-	-	-	-	+
430	-	NA	NA	+	-	-	-	NA
440	-	-	+	-	-	-	-	NA
450	-	-	-	-	+	-	-	-
460	-	-	+	-	-	NA	-	NA
470	-	-	+	-	-	-	-	NA
480	-	NA	NA	-	-	-	NA	NA
490	-	+	+	-	-	-	-	NA
500	+	-	-	-	-	-	-	NA

Table S2. Association between IAV infection and nasal secretion at week 2.

**P-Value**	**Week 2**				
**1**	**Nasal Discharge**				
			+	-	**Total**
The Fisher exact test statistic value is 1. The result is not significant at p < .05	**IAV**	+	2	0	2
		-	31	13	44
		**Total**	33	13	**46**

Table S3. Association between IAV infection and nasal secretion at week 4.

**P-Value**	**Week 4**				
**0.1053**	**Nasal Discharge**				
			+	-	**Total**
The Fisher exact test statistic value is 0.1053. The result is not significant at p < .05.	**IAV**	+	2	11	13
		-	0	26	26
		**Total**	2	37	**39**

Table S4. Association between IAV infection and conjunctivitis at week 2.

**P-Value**	**Week 2**				
**1**	**Conjunctivitis**				
			+	-	**Total**
The Fisher exact test statistic value is 1. The result is not significant at p < .05	**IAV**	+	1	1	2
		-	19	25	44
		**Total**	20	26	**46**

Table S5. Association between IAV infection and conjunctivitis at week 4.

**P-Value**	**Week 4**				
**1**	**Conjunctivitis**				
			+	-	**Total**
The Fisher exact test statistic value is 1. The result is not significant at p < .05.	**IAV**	+	1	12	13
		-	3	23	26
		**Total**	4	35	**39**

Table S6. Nasal swabs selected for whole genome sequencing (WGS).

Pig ID	Week 4	Week 5	Week 6	Week 8	Week 22	Total
90	WGS					1
210				WGS		1
250	WGS			WGS		2
290			WGS			1
310	WGS					1
380		WGS			WGS	2
420					WGS	1
440	WGS					1
490	WGS					1
**Total**	5	1	1	2	2	11
